# Supplementary figures and images for: The Effect of Different Glucose Concentrations on the Antiproliferative Activity of Metformin in MCF-7 Breast Cancer Cells
Source: Pharmaceutics. 2023 Aug 23;15(9):2186. doi: 10.3390/pharmaceutics15092186 (PMC10537756; doi:10.3390/pharmaceutics15092186)

Gel 1 - Alpha-Tubulin

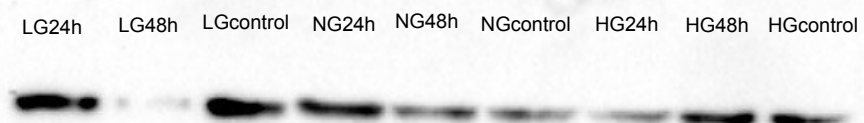

Gel 2 - BCL-2

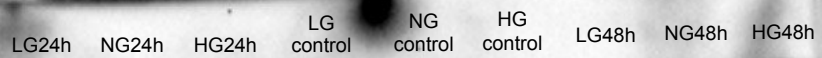

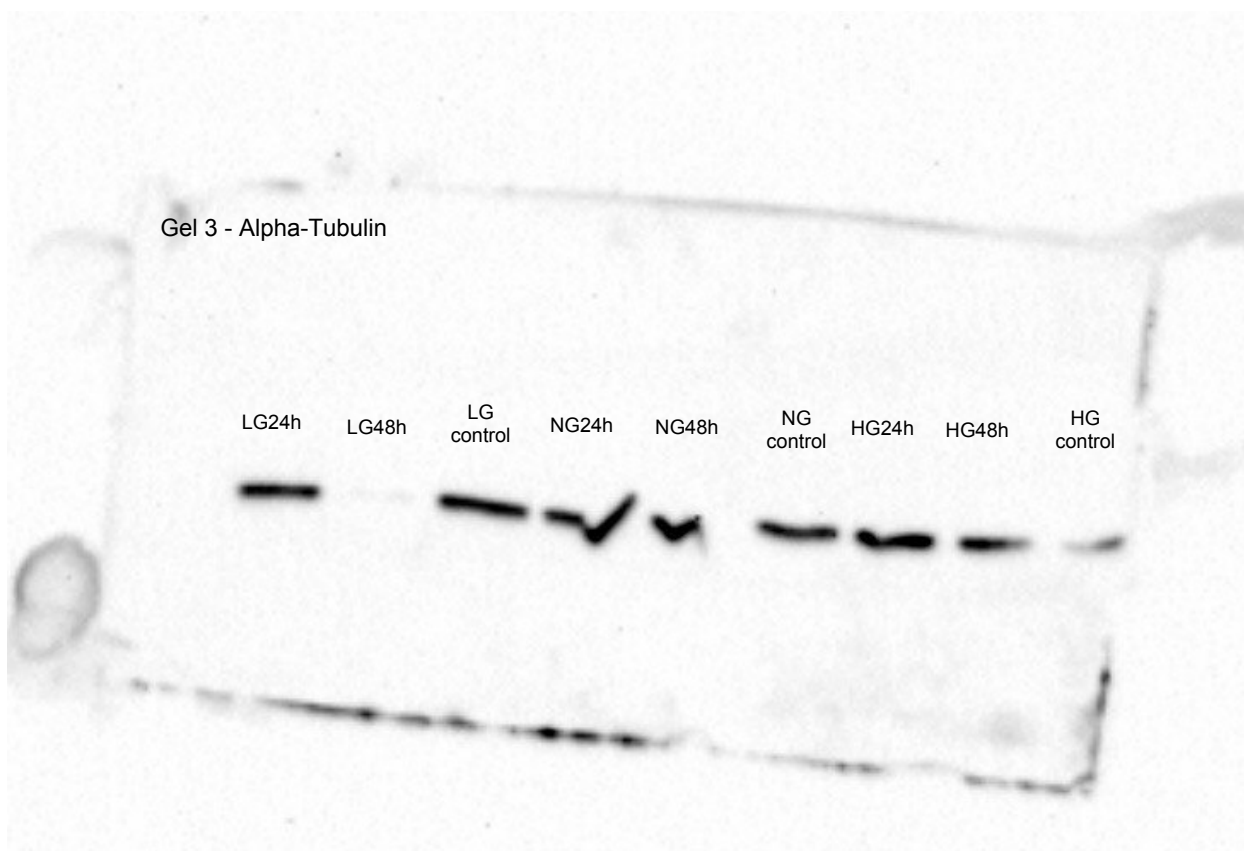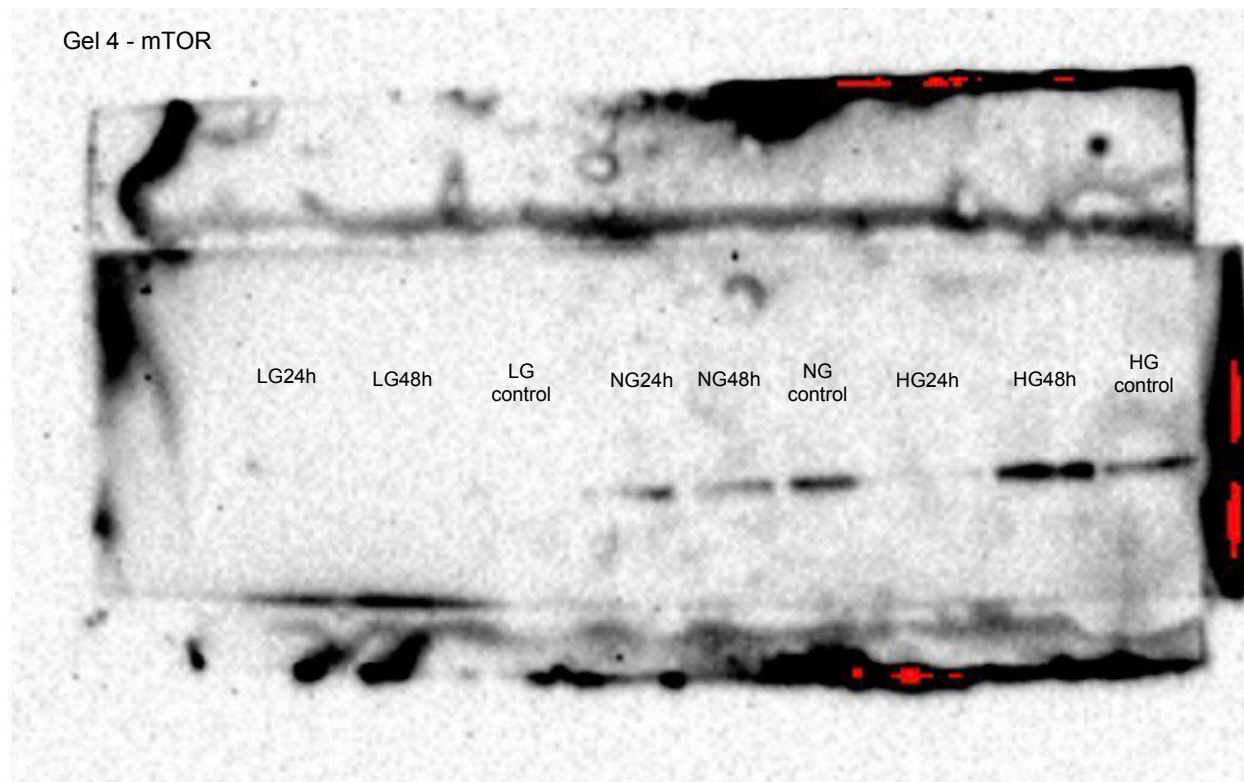

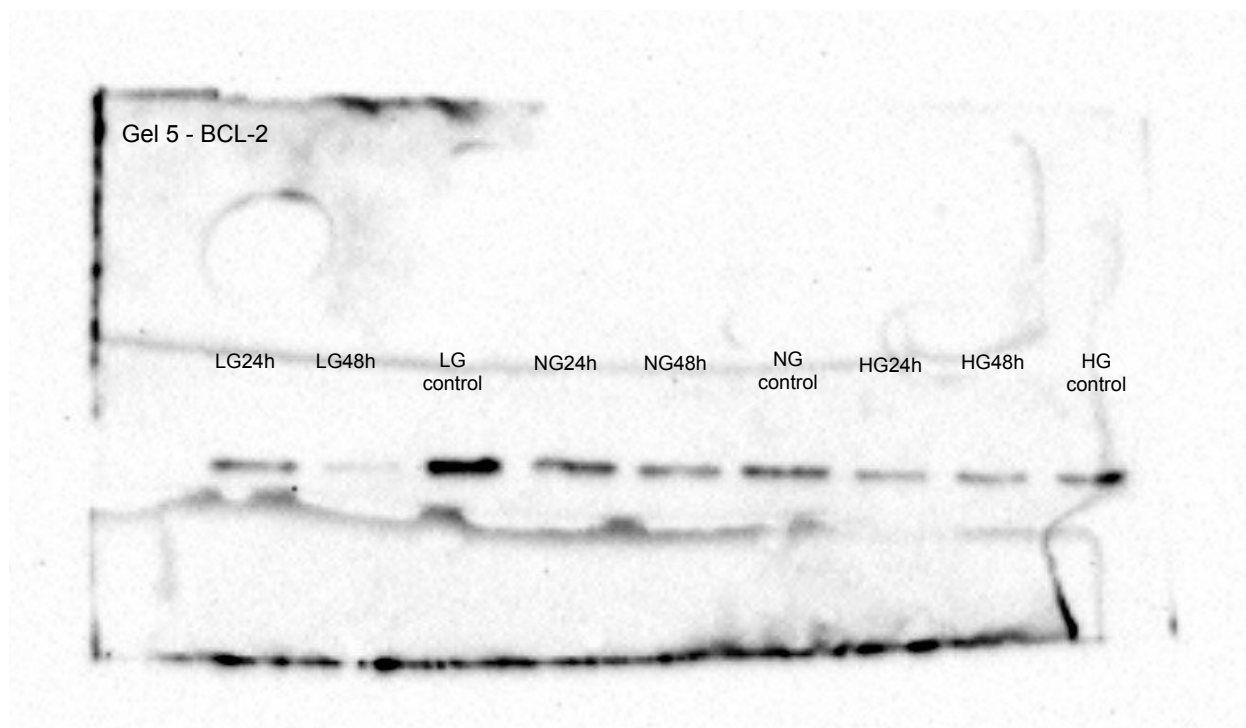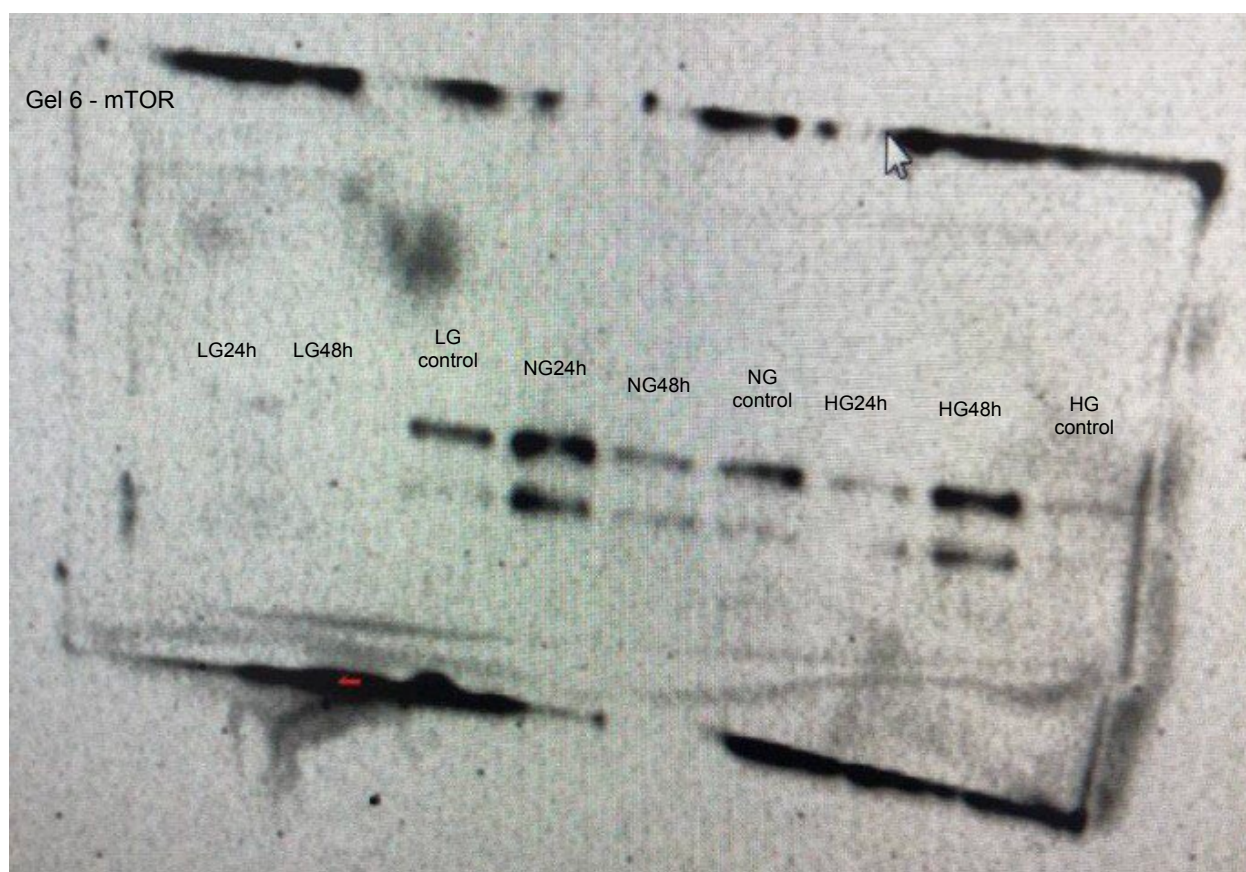

Supplement: Supplementary file 1 [file pharmaceutics-15-02186-s001.zip › pharmaceutics-2553169-supplementary.pdf]
